# Preparation and biomedical application of light-responsive hydrogels based on natural products

**DOI:** 10.3389/fphar.2025.1714907

**Published:** 2025-12-03

**Authors:** Xinyi Fan, Haihua Shang, Leyuan Ji, Shitang Ma, Maoliang Liao

**Affiliations:** 1 College of biological and pharmaceutical engineering, West Anhui University, Lu’an, China; 2 Anhui Engineering Laboratory for Conservation and Sustainable Utilization of Traditional Chinese Medicine Resources, West Anhui University, Lu’an, China; 3 Tianjin Key Laboratory of Radiation Medicine and Molecular Nuclear Medicine, Institute of Radiation Medicine, Peking Union Medical College and Chinese Academy of Medical Sciences, Tianjin, China; 4 College of Traditional Chinese Medicine, Anhui University of Chinese Medicine, Hefei, China; 5 Anhui Province Key Laboratory of Bioactive Natural Products, Hefei, China; 6 Anhui Provincial Common Technology Research Center for Modern Chinese Medicine Industry, West Anhui University, Lu’an, China

**Keywords:** Fundamental characteristics, natural products, light-responsive hydrogels, physical and chemical crosslinking, biomedical applications, quality evaluation frameworks

## Abstract

This article systematically reviews the light-responsive hydrogels based on natural products, with a focus on their extensive applications in the field of biomedicine. Regarding the preparation methods, the article elaborates on the physical self-assembly, chemical crosslinking agent method, photocrosslinking method, and free radical polymerization method. The biomedical application section introduces their applications in drug release and wound healing based on the mechanisms of photochemical reactions, photoisomerization reactions, and photothermal reactions. In terms of quality evaluation frameworks, the natural light-responsive hydrogels evaluation frameworks is summarized, and the shortcomings of the existing evaluation frameworks are analyzed. Finally, the article summarizes its application potential and development bottlenecks, looks forward to future research directions, and proposes targeted development suggestions, thereby promoting its wider applications in deep disease treatment and personalized medicine.

## Graphical Abstract

**Figure:**
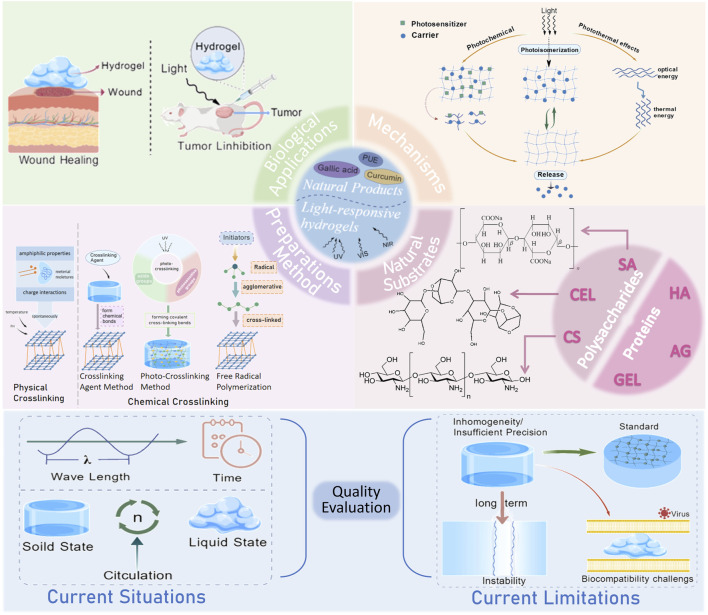

## 1 Introduction

Hydrogels are an excellent type of biomaterial, receiving significant attention in the field of biomedical applications. Light-responsive hydrogels are a class of three-dimensional network-like polymer materials with intelligent light-responsive properties. They are composed of a substrate polymer framework and photoreactive functional units. Through mechanisms such as light cross-linking, light isomerization, and light degradation (Ji et al., 2020), their structures and properties can be precisely controlled under specific light exposure. Due to their advantages such as controllable time and space (Liu et al., 2019), mild stimulation (Marambio et al., 2025), and rapid response (Pai et al., 2024), they are widely used in biomedical fields such as drug controlled-release (Giacolone et al., 2024), tissue engineering (Bai et al., 2024) and flexible electronics (Hu et al., 2024; Xie et al., 2021).

Light-responsive hydrogels based on natural products are materials formed by using natural polymers such as hyaluronic acid (HA) (Wang et al., 2024) and gelatin (Gel) (Liang et al., 2023) as the substrate, and introducing photoreactive components through chemical modification or physical combination. They inherit the common photoreactive response characteristics of light-responsive hydrogels, and due to the excellent biocompatibility, controllable biodegradability (Singh et al., 2025), and inherent biological activity of the natural substrate, they have become core application materials in biomedical scenarios such as wound repair (Yang et al., 2025), 3D bioprinting (Kamal et al., 2023), and drug carriers (Yang et al., 2020).

This article reviews the preparation methods of light-responsive hydrogels based on natural products and the latest progress in their biomedical applications. Many hydrogels with excellent functions have been prepared through various means. The article also looks forward to further research directions.

## 2 Fundamental characteristics of natural products-based light-responsive hydrogels

### 2.1 Light-responsivity

Based on their response mechanisms, hydrogels can be classified into conventional hydrogels and smart hydrogels. Light-responsive hydrogels are a subclass of smart hydrogels, designed by introducing light-responsive materials. They can trigger structural or conformational changes by adjusting conditions such as light intensity, wavelength, or exposure time, thereby achieving controlled drug release in space and time (Kaith et al., 2021; Tang et al., 2023). Light is a non-invasive and easily accessible stimulus source with high focal and flexibility, and it can achieve precise control of drug release time and location without physical contact (Hua et al., 2024; Xing et al., 2022). Unlike stimuli such as pH value or temperature, light can be externally manipulated, which helps to enhance the targeted effect of treatment and reduce toxicity. light-responsive hydrogels based on natural products use natural products as a safe substrate and photoreactivity as a precise control method.

### 2.2 Characteristics of natural products

One of the common classification methods for hydrogels is to categorize them based on their synthetic materials into natural products-based hydrogels and synthetic hydrogels (Dodda et al., 2023). The protein-based category includes collagen (CO), Gel, fibrin, etc. (Ma et al., 2020); the polysaccharide-based category includes agarose (AG) (Sacco et al., 2024), chitosan (CS) (Alven and Atim Aderibigbe, 2020), sodium alginate (SA) (Lueckgen et al., 2018), cellulose (CEL) (Pan et al., 2023), and HC (Maiz-Fernández et al., 2022). Commonly used materials for synthesizing hydrogels are usually high-molecular-weight materials, such as polyacrylamide (Afuwape and Hill, 2021), polyvinyl alcohol, and polyamidoamine.

### 2.3 The advantages of natural light-responsive hydrogels

#### 2.3.1 Reliable and easily accessible sources

From the perspective of raw material sources, Gel is derived from the skin and bones of animals, HA exists in the dermis of the human body (Valachová et al., 2024), CEL comes from the cell walls of plants, and CS comes from the shells of shrimp and crabs (Samiraninezhad et al., 2023). These raw materials either directly originate from biological organisms or are extracted from plants and microorganisms. Their chemical structures are highly compatible with the natural components within biological organisms. Synthetic hydrogels mostly rely on high-molecular synthetic materials and require complex chemical synthesis processes. Natural product raw materials, on the other hand, do not require complex chemical synthesis processes and can reduce the residue of toxic monomers. Different sources, extraction methods, and collection times can all have an impact on the structure or activity of photosensitive components, which leads to the diversity of natural hydrogels and also becomes a factor that needs to be considered during the preparation process (Zhang et al., 2018).

#### 2.3.2 Superior biocompatibility

In terms of biocompatibility, most natural substances can be enzymatically decomposed or degraded in the physiological environment of the body. The degradation products, such as monosaccharides and small molecule peptides, can be absorbed and metabolized by the human body, avoiding the risk of foreign matter accumulation, and laying a safe foundation for its application in scenarios such as *in vivo* drug delivery and wound repair (Meng et al., 2025). They not only overcome the defect of poor biocompatibility of synthetic photo-responsive hydrogels, but also make up for the deficiency of traditional natural hydrogels in precise functional control.

#### 2.3.3 Adjustable properties

By adjusting the concentration of natural products, the mechanical strength of the hydrogel can be regulated. Bian et al. (2013) found that when the concentration of HA increased from 1% to 5% (w/v), the crosslinking strength of the hydrogel also increased. Liu C. et al. (2024) found that as the CS content increased to 20%, 40%, and 50%, the porosity increased to approximately 40%, 42%, and 55%, respectively; as the CS content increased, the swelling performance also improved, indicating a more porous structure. Alipal et al. (2021) also believed that the Gel strength and yield of processed gelatin were largely related to its pretreatment and extraction methods.

Natural hydrogels are mostly crosslinked through natural intermolecular forces, with a low crosslinking density, and are softer. They are prone to deformation and rupture under external force, but due to these characteristics, they are more suitable for photothermal hydrogel drug delivery (Xie et al., 2024) than synthetic hydrogels. Photothermal hydrogels mostly need to be delivered into the body through injection. When the hydrogel is injected, the network structure is temporarily disrupted and the viscosity decreases under the push of the syringe. However, natural hydrogels, due to their suitable mechanical strength, are more easily pushed out by the syringe after entering the body. Not only can the network structure be restored through molecular interaction, but also the phototoxicity can be utilized to achieve re-crosslinking. In contrast, the greater mechanical strength of synthetic hydrogels makes it difficult for the disrupted network structure to be restored during the injection process.

Thus, natural products offer a versatile and biocompatible platform for constructing advanced light-responsive hydrogels, which will be systematically reviewed in this article. The unique properties of natural products make them highly suitable for the construction of light-responsive hydrogels, thereby enabling them to play a more effective role in biomedical applications such as drug delivery and tissue engineering.

## 3 Preparation method of natural product-based light-responsive hydrogels via physical and chemical crosslinking

The promising biomedical functionalities of light-responsive hydrogels based on natural products are contingent upon the successful fabrication of their three-dimensional network structures. The choice of preparation strategy is paramount, as it directly governs key properties such as mechanical strength, degradation kinetics, and the efficiency of the light-response. This section critically reviews and compares the principal methods employed to construct these hydrogels, which are broadly categorized into physical crosslinking and chemical crosslinking approaches, each offering distinct advantages and limitations.

### 3.1 Physical crosslinking

Physical crosslinking is achieved through weak interactions such as intermolecular hydrogen bonds and hydrophobic forces to form a network, and it has the characteristic of mild action. This method can fully utilize the interactions inherent in the natural products themselves. The following is an introduction to the common self-assembly methods.

Self-assembly is a typical method of physical cross-linking. By taking advantage of the amphiphilic properties and charge interactions of the material molecules, it can spontaneously arrange and form an ordered three-dimensional network under the influence of temperature and pH changes.

**FIGURE 1:**
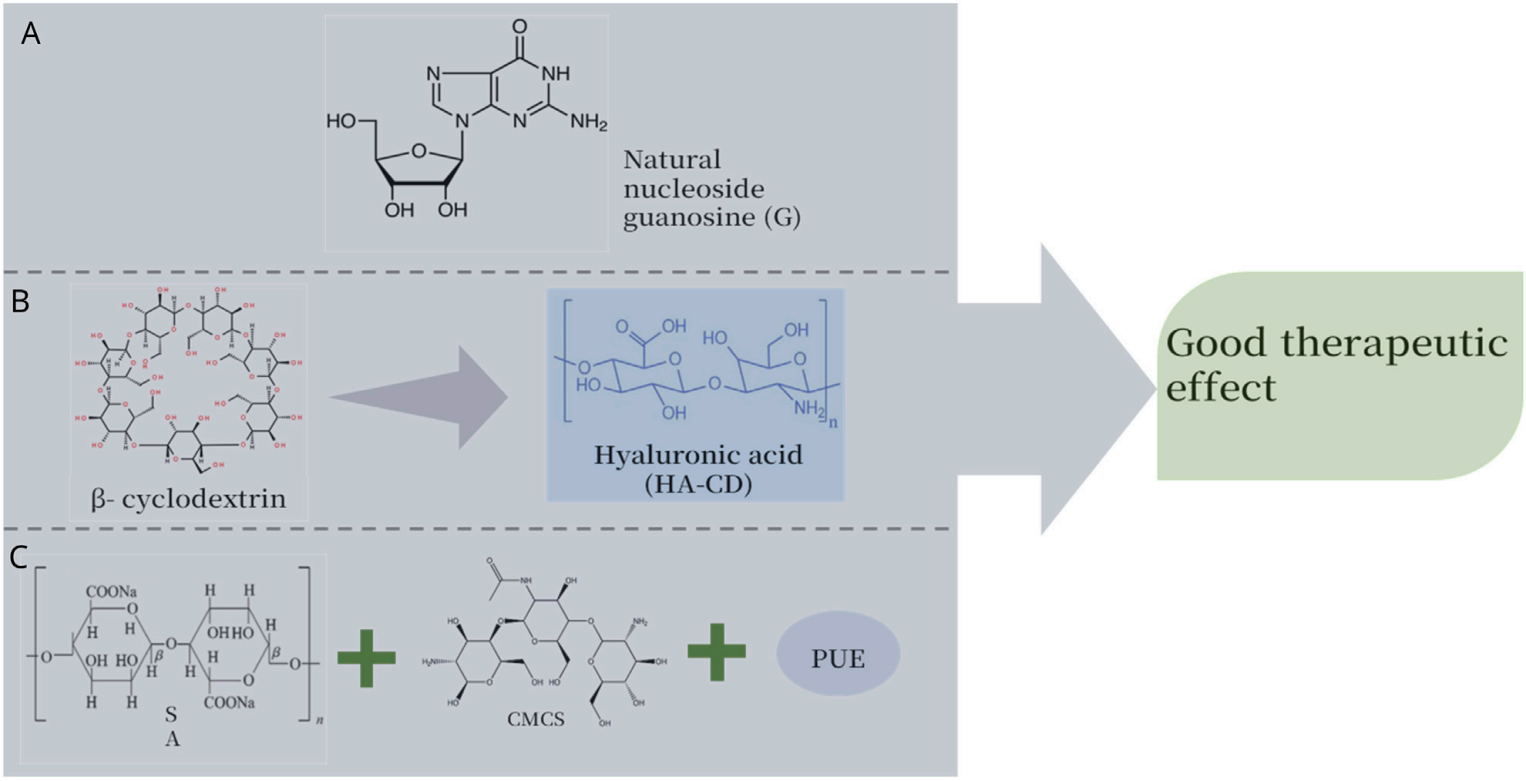
The hydrogels prepared by self-assembly method, namely, natural product photosensitive hydrogels, have better therapeutic effects compared to ordinary hydrogels. **(A)** Guanosine hydrogel (Lv et al., 2025). **(B)** β-cyclodextrin modified HA-cd hydrogel (Xu et al., 2025). **(C)** CMCS/OSA/MPDA@PUE hydrogel prepared with CMCS, SA, and PUE as raw materials (Zhai et al., 2025).

In the G@GQD light-responsive hydrogel prepared by Lv et al. (2025), guanosine (G), a natural nucleoside derived from biological systems, self-assembles into G tetramers through intermolecular hydrogen bonds. The ribose diol groups of G form dynamic borate ester bonds with the boronic acid of 2-formylbenzylboronic acid (2-FPBA), and then a three-dimensional fiber network is constructed through longitudinal π-π stacking. At the same time, graphene quantum dots (GQDs) are physically encapsulated and electrostatically bound within it, ultimately self-assembling into a stable hydrogel. Xu et al. (2025) utilized the host-guest interaction between HA modified by β-cyclodextrin (HA-CD) and HA modified by ferrocene (HA-Fc) to achieve self-assembly, forming supramolecular hydrogels (HACF). At the same time, they encapsulated photodynamic agent indocyanine green (ICG) and anti-cancer drug DOX within it, providing a solution for the prevention and precise treatment of postoperative recurrence of superficial tumors such as breast cancer. Zhai et al. (2025) prepared CMCS/OSA/MPDA@PUE by using carboxymethyl chitosan (CMCS), SA, and puerarin (PUE). This material, composed of multiple natural products, can rapidly release PUE through photothermal reaction, and it can be applied in promoting wound healing. H&E staining showed that the hydrogel had good biocompatibility and efficacy (Figure 1).

Although the self-assembled hydrogels have the drawback of relatively weak mechanical properties, their convenient and mild reaction conditions make them promising for achieving a good combination with hydrogels prepared from natural products. In addition, blending and hydrothermal methods are also physical approaches used for the preparation of natural photosensitive hydrogels.

### 3.2 Chemical crosslinking

Chemical crosslinking builds a network structure by forming stable covalent bonds between molecules. When it comes to light-responsive hydrogels based on natural products, this method utilizes the reactive groups present in natural products to form covalent crosslinks. Compared with physical crosslinking, the resulting materials have higher mechanical strength and stronger structural stability, but the process is usually irreversible.

#### 3.2.1 Crosslinking agent method

The crosslinking agent method involves adding crosslinking agents to hydrogels to form chemical bonds between molecules, thereby obtaining the network structure of the hydrogel. Common crosslinking agents include N,N'-methylene diacrylamide (MBA), PEGDA, 4-azobenzoic acid (AzBA), and benzophenone (BP), etc.

**FIGURE 2:**
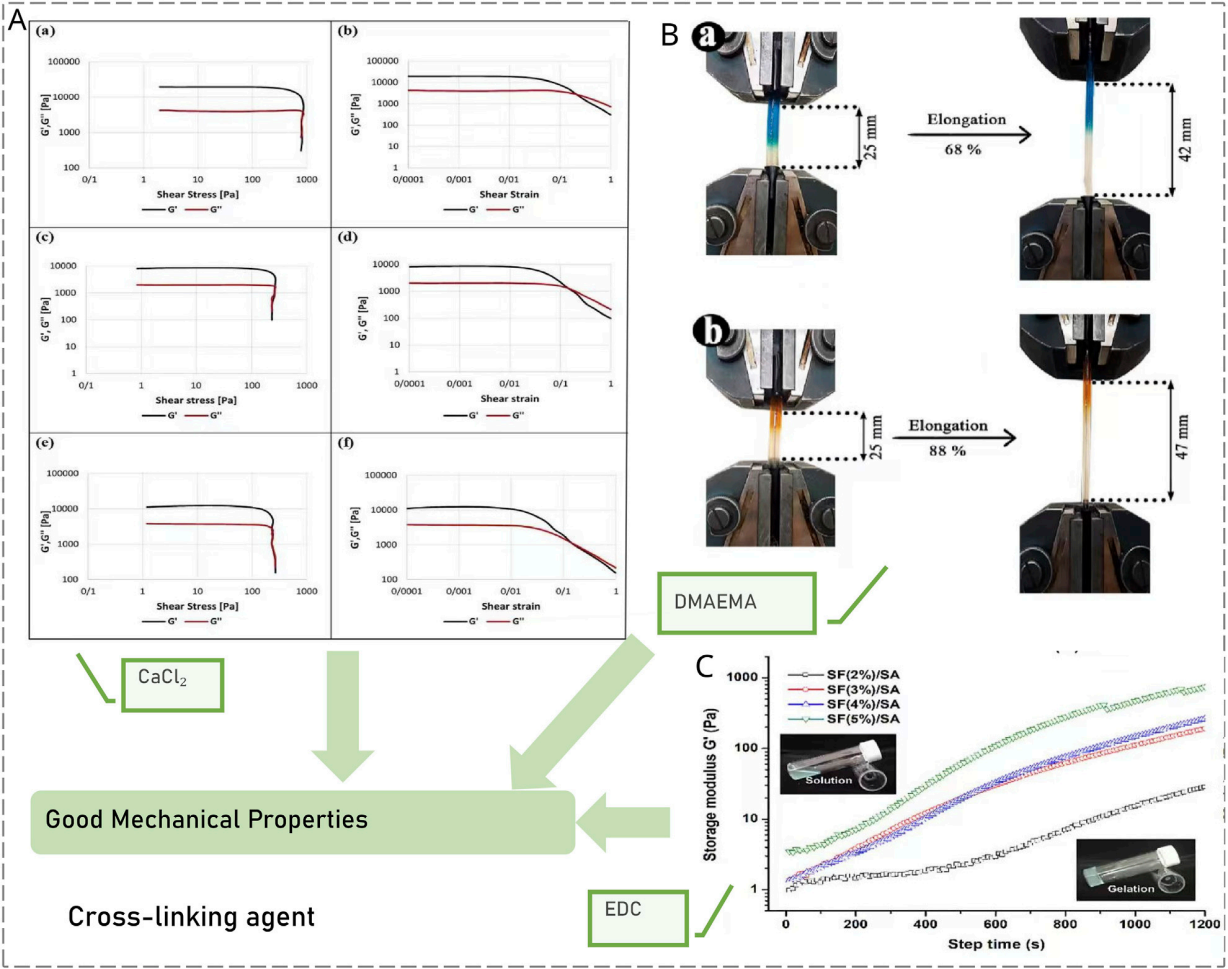
The water gels prepared by the crosslinking agent method have excellent mechanical properties. **(A)** Calcium chloride is used as the crosslinking agent. All water gels are mechanically stable (Bahraminasab et al. 2023). **(B)** DMAEMA and others are used as crosslinking agents. The tensile properties are good (Pourbadiei et al. 2023). **(C)** EDC is used as the crosslinking agent. In all cases, the water gels exhibit high elasticity (Niu et al. 2021).

Bahraminasab et al. (2023) prepared alginate - gelatin composite hydrogel using Gel and SA as raw materials and CaCl_2_ as the crosslinking agent. It is non-toxic, non-immunogenic and will not cause cytotoxic reactions. Experimental verification shows that when the proportion of gelatin is 5%, the 7-day proliferation rate of cells is significantly higher than that of other groups, proving that it supports cell survival. Light-responsive gelatin-based self-healing interpenetrated network (IPN) hydrogel prepared by Pourbadiei et al. (2023) Under a 300W xenon lamp, Fe^3+^ is reduced to Fe^2+^ using crosslinking agents such as dimethyl aminoethyl methacrylate (DMAEMA), and it has a good self-healing function. The SF/SA@ICG composite hydrogel prepared by Niu et al. (2021) uses 1-ethyl-3-(3-dimethylamino-propyl) carbodiimide (EDC) as the crosslinking agent. Under 808 nm near-infrared (NIR) light irradiation, it can achieve photothermal conversion. At an ICG concentration of 8 μg/mL, irradiation for 1 min can increase the temperature of the hydrogel by approximately 8 °C, thereby effectively triggering the on-demand release of the encapsulated bovine serum albumin (BSA) and tetracycline hydrochloride (TH) (Figure 2).

The hydrogels prepared by the crosslinking agent method have the advantages of stable materials and wide application range. When natural products are involved, this method can utilize the reactive groups in natural products like polysaccharides or proteins to form covalent crosslinks with the help of crosslinking agents. However, problems such as high reaction conditions requirements, residual crosslinking agents, cost and environmental protection still exist.

#### 3.2.2 Photo-crosslinking method

Unlike the crosslinking agent method, the photo-crosslinking method requires the addition of a photosensitive crosslinking agent to achieve the effect of photosensitive crosslinking. The photopolymerization method utilizes ultraviolet light and visible light to trigger chemical reactions of photosensitive groups such as acrylate groups (Walther et al., 2018) and azide groups (Buwalda, 2024), rapidly forming covalent cross-linking bonds, and common natural photosensitizers include curcumin (Li W.-X. et al., 2023) and indocyanine green (ICG), among others. It has the advantages of controllable reaction, high efficiency, and the ability to achieve spatial-selective cross-linking, which is beneficial for precisely constructing hydrogel networks based on natural products.

**FIGURE 3:**
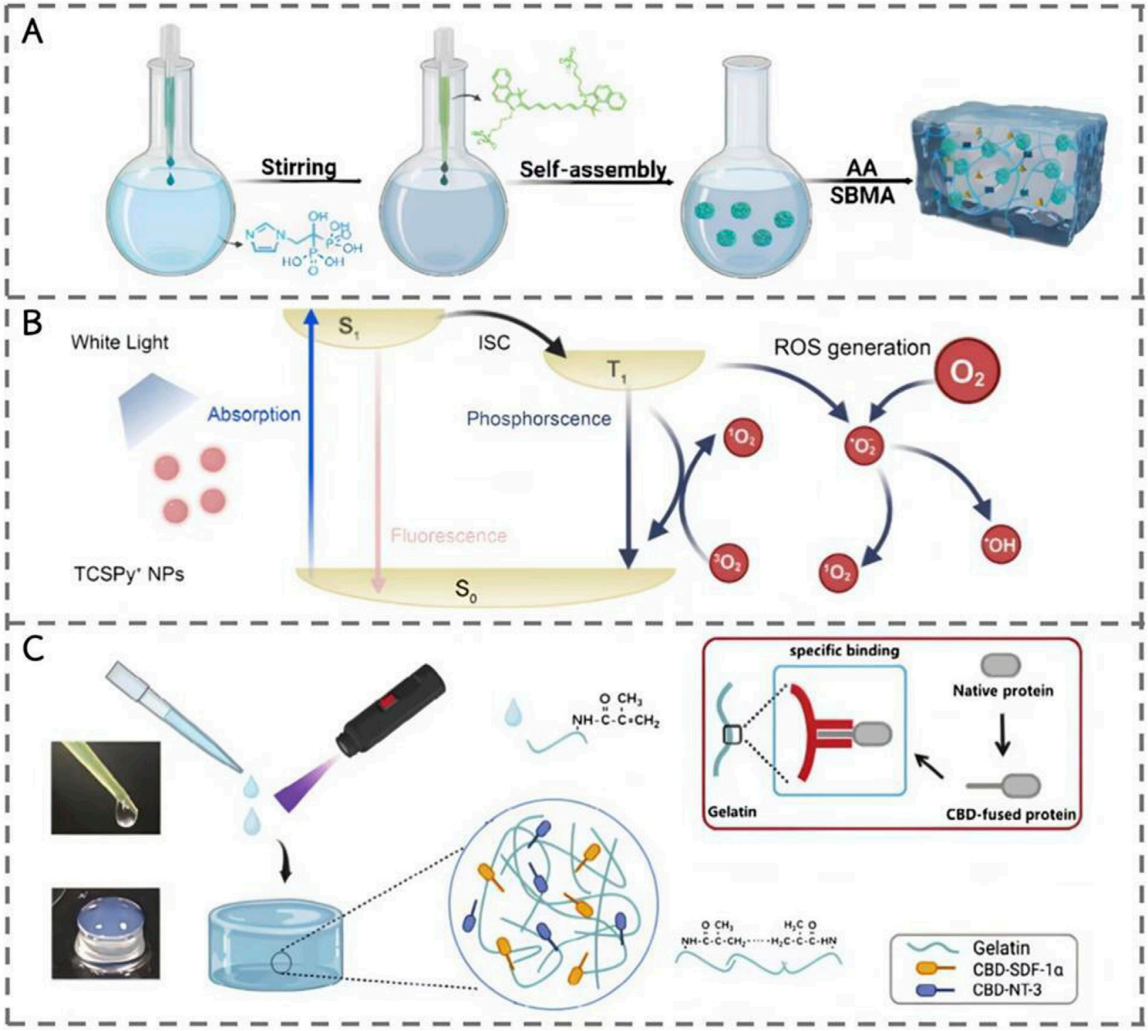
The design principle of hydrogels. **(A)** Synthesis of the PAS/ICG-Cu@ZOL hydrogel dressing (Ren et al., 2025). **(B)** The schematic of a Jablonski diagram (Liu et al., 2025). **(C)** Schematic diagram showing the preparation of light responsive hydrogel scaffold and the design ofSDF1*α* and NT3 with collagen-specific binding ability (Liu D. et al., 2024).

Ren et al. (2025) added ICG as a photoinitiator to trigger the polymerization reaction of AA and SBMA monomers, forming a polyacrylic acid-methacrylic acid copolymer sulfobetaine (PAS) hydrogel matrix, thereby achieving stable loading of the ICG-Cu@ZOL complex in the matrix. In the research using lithium phosphonate (LAP) with phenyl (2,4, 6-trimethylbenzoyl) as the photoinitiator. Liu et al. (2025) Under the irradiation of 405 nm ultraviolet light, they initiated the cross-linking reaction between the double bonds of methacryloyl (GelMA) and polyethylene glycol diacrylate (PEGDA) in gelatin, and eventually formed a hydrogel network with specific structure and properties. Liu D. et al. (2024) then, after irradiation with 395 nm ultraviolet light for 10–20 s, specific binding occurred between the prepared CBD-fused protein and methacrylic acid gelatin molecules to form a double-modified light-responsive hydrogel (Figure 3).

By the method of photo-crosslinking, hydrogels with specific structures and properties can be formed, and these hydrogels can stabilize the loaded complexes. However, there may also be problems such as multiple restrictions on crosslinking conditions and uneven crosslinking.

#### 3.2.3 Free radical polymerization

Free radical polymerization is a method that involves initiating the polymerization of monomers through free radicals and forming a cross-linked structure. Usually, initiators such as peroxides and azo compounds are used to generate free radicals, which then initiate the growth of the monomer chain and form a three-dimensional network through the cross-linking points.

**FIGURE 4:**
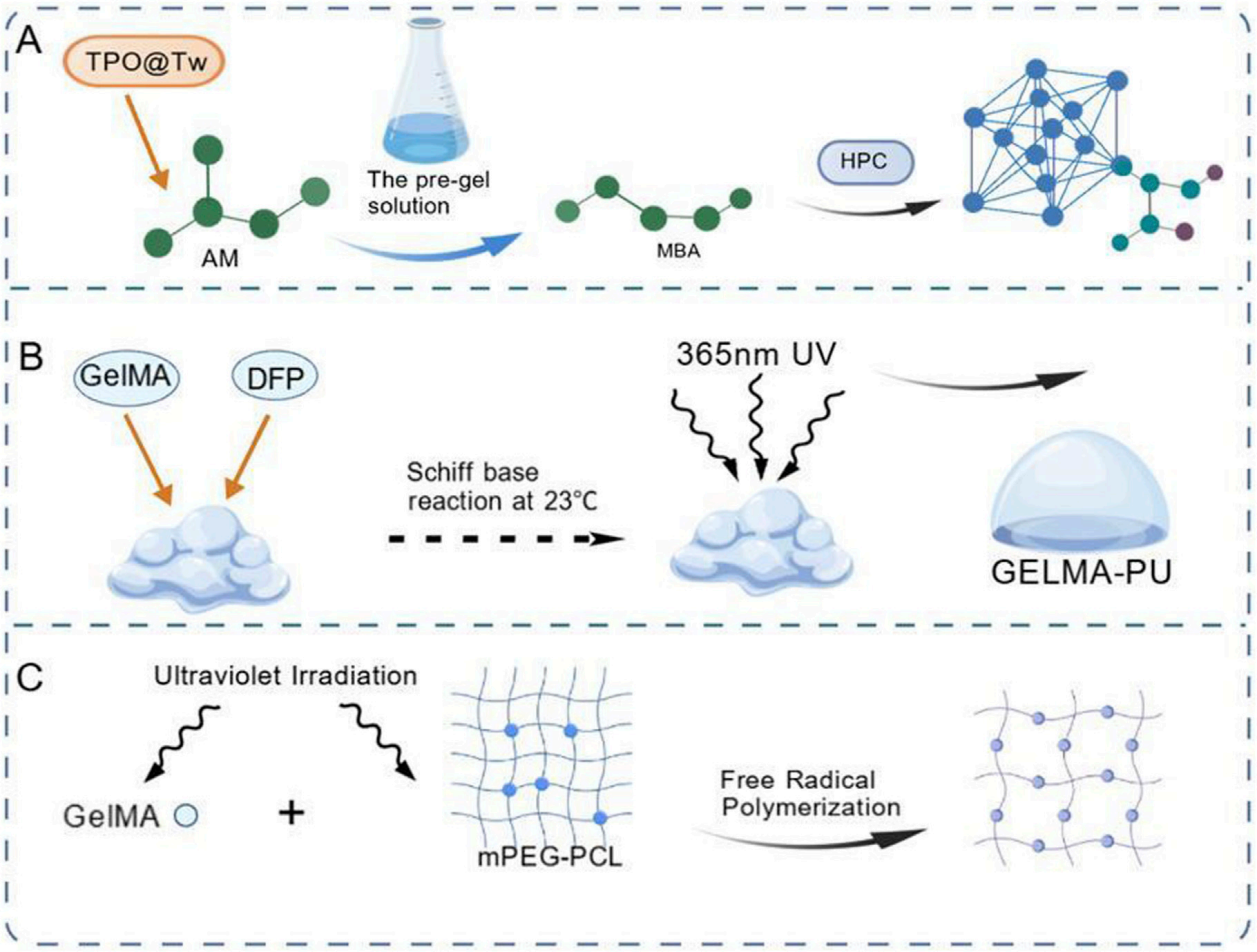
The principle of free radical polymerization. **(A)** TPO@Tw initiates AM polymerization, followed by MBA cross-linking and HPC intercalation to form a dual-network hydrogel (Zhang et al., 2023). **(B)** GelMA and DFPU undergo Schiff base reaction and free radical polymerization initiated by ultraviolet light (Cheng and Hsu, 2023). **(C)** GelMA and mPEG-PCL nanoparticles were subjected to free radical polymerization under ultraviolet light (Li et al., 2020).

Zhang et al. (2023) initiated the polymerization of AM monomers in pre-gel solution to form linear chains by means of free radicals generated by TPO@Tw. Meanwhile, MBA cross-links the linear chains into a three-dimensional network, and hydroxypropyl cellulose (HPC) is embedded in the network to form a dual-network structure, achieving complete photopolymerization. The GELMA-PU hydrogel prepared by Cheng and Hsu (2023) was initially cross-linked and formed by proportionally mixing GelMA modified from pig gelatin with dialdehyde functionalized polyurethane (DFPU) through Schiff base reaction at 23 °C. After being further subjected to 365 nm UV light irradiation (free radical polymerization initiated by photoinitiator VA-086) for secondary cross-linking and strengthening, it possesses self-healing, 3D printing, biocompatibility and other properties. Li et al. (2020) used gelatin methacrylate (GelMA) and artemisinin-supported mPEG-PCL nanoparticles as raw materials. They were subjected to ultraviolet light irradiation to initiate free radical polymerization reactions and cross-linking to form hydrogels. This hydrogel has therapeutic potential for gel-induced hearing loss and lays the foundation for clinical research (Figure 4).

Free radical polymerization can be applied in wound dressings and clinical use to provide effective assistance because it can effectively prevent ultraviolet light from damaging cells. However, there are also problems such as free radical toxicity and oxygen inhibition of polymerization.

## 4 Biomedical applications driven by light-response mechanisms

The biomedical applications of these hydrogels are fundamentally governed by their light-response mechanisms, which include photochemical reactions, photoisomerization, and photothermal effects. Through the contents under different mechanisms, the application of natural product light-responsive hydrogels will be introduced. The core feature of light-responsive hydrogels lies in the addition of photosensitive groups to the polymer network, enabling structural and conformational changes upon light exposure. These changes include the disruption of cross-links, expansion, contraction or degradation, which directly regulate drug release (Liu S. et al., 2024).

### 4.1 Photochemical reactions

This section focuses on photochemical reactions, which are further divided into three types: photooxidation, photopolymerization, and photolysis. It then introduces the chemical processes, key substances, and their effects on the structure of drug carriers for each of these reaction types.

#### 4.1.1 Photooxidation

This reaction requires the participation of molecular oxygen, photosensitive compounds and excitation light. After the photosensitizer absorbs photons, it undergoes energy level transition and generates reactive oxygen species (ROS) through I-type or II-type electron transfer. The reactive oxygen can oxidize the components of the nanocarriers such as unsaturated lipids, destroying the carrier structure and forming channels or micelles, thereby triggering the release of the drugs.

In the acute phase (3–7 days) after spinal cord injury, ROS are released in the injury area. The diselenide bonds in F10@MS prepared by Zhang K. et al. (2025) are oxidized and broken by the high concentration of ROS in the injury area, leading to the disintegration of the mesoporous silica nanoparticles structure and the subsequent release of F10 originally encapsulated within the mesopores. This achieves the precise synchronization of drug release with the pathological process of spinal cord injury. The photosensitizer perylene diimide dopamine (PBI-DOPA) in the hydrogel prepared by Rostaminejad et al. (2023) was excited to the triplet state under the irradiation of 650 nm laser and 530 nm LED light. Through energy transfer, it underwent photochemical reactions with oxygen molecules, generating singlet oxygen and other highly cytotoxic substances. Heidarpour et al. (2020) prepared Ag/AgCl@Al-CMC and AgCl@Fe-CMC. Under the irradiation of 400 nm and 420 nm visible light, the AgCl in the hydrogel beads was excited to generate photogenerated electron-hole pairs. Ag^0^ or Fe^3+^ captured the electrons to inhibit the complexation. The photogenerated holes directly oxidized rhodamine B or reacted with OH^−^ to form ·OH. The ·OH served as the main active species to oxidize and degrade rhodamine B, achieving the removal of pollutants through photolysis. Wang Y. et al. (2025) prepared Ga2O3NPs hydrogel. When exposed to xenon lamp, this hydrogel could generate reactive ROS, damage the bacterial cell membranes and induce the leakage of nucleic acids to kill multidrug-resistant bacteria including *Escherichia coli* and *Staphylococcus aureus*, all of which demonstrated excellent antibacterial effects.

Photocatalytic reactions have the characteristics of wide application range, environmentally friendly reaction conditions, and strong controllability. When natural products are involved, these reactions can utilize the inherent light-responsive or catalytic properties of natural substances. However, they also have problems such as strong dependence on light and susceptibility to interference in the reaction system, and these issues may be further influenced when natural products with complex compositions are used.

#### 4.1.2 Photolysis

Photolysis (photodecomposition) is the most commonly used photochemical reaction method. By introducing photodegradable linkers into polymers or lipids, such as o-nitrobenzyl groups and coumarin dimers, when exposed to light of an appropriate wavelength, the linkers undergo chemical bond cleavage, destroying the structure of the nanocarriers and thereby promoting drug release. Different photodegradable linkers have different characteristics. The o-nitrobenzyl group is commonly used as a photoactive protecting group, and when combined with natural-product scaffolds, it can endow the system with both light-responsiveness and biocompatibility. While coumarin derivatives have more flexibility in the temporal and spatial control of drug release due to their reversible photo-dimerization and photodecomposition properties.

The o-NB (a nitrobenzyl derivative) in the MeHA-NB hydrogel prepared by Li Z.-Y. et al. (2023) undergoes chemical bond cleavage under 254 nm ultraviolet light irradiation, causing the three-dimensional cross-linked network of the hydrogel to disintegrate, resulting in its degradation from a solid state to a washable and removable liquid-like state. The photolytic product 1-(2-nitrophenyl) ethyl-1-ketone still forms a covalent bond with the MeHA polymer chain and will be completely washed away along with the liquid-like substance, avoiding the spread of the product on the wound surface and causing adverse effects. This creates a clean environment for the subsequent *in situ* gelation of the new dressing. The core characteristic of the light-enzyme coupled hydrogel platform prepared by Hu et al. (2023) is “light-switch redox reversibility”: When exposed to blue light, dihydrolipoamide dehydrogenase (DLD) is activated, generating reactive ROS such as hydroxyl radicals (·OH) through electron transfer, thereby initiating an oxidation reaction to achieve antibacterial effects; without light exposure, DLD initiates an antioxidant cycle, removing ROS by extracting protons and electrons from reducing agents, and initiating a reduction reaction to alleviate oxidative stress, thereby achieving bidirectional regulation of the oxidative-reductive microenvironment of the wound site. An et al. (2024) demonstrated that 365 nm UV light irradiation could cause the photolysis of the o-NB group and the disruption of the hydrophilic environment. At the same time, an increase in temperature could weaken the double hydrogen bonds, resulting in the loosening of the hydrogel network. Together, these mechanisms enabled the controlled release of rifampicin and doxorubicin.

The reason why photolysis method is widely used is that its reaction conditions are mild and highly adjustable, especially when it comes to the photodegradable linkers derived from natural products. However, there are still some potential problems, such as insufficient photostability and stability, which may become more prominent when dealing with natural products with complex molecular structures.

#### 4.1.3 Photopolymerization

Photopolymerization is a mechanism by which, under the illumination of light, molecules with unsaturated bonds in the carrier undergo polymerization and cross-linking, causing the structure of the carrier to shrink or form a porous network, thereby disrupting the stability of the carrier and facilitating drug release.

Ku et al. (2022) using PEGDA as the base material, nano-silicate sheets, phenyl-2,4,6-trimethylbenzoyl phosphonic acid lithium (LAP), and iodine-containing contrast agents were added to prepare a low-viscosity, shear-thinning and radiopaque photoinitiated hydrogel polymer embolic agent; when irradiated by a 405 nm laser, LAP activates and initiates the cross-linking and curing of PEGDA, and placing the optical fiber in a balloon catheter containing 1% fat emulsion can optimize the light distribution to achieve complete curing; this embolic agent was injected into the wide-necked aneurysm through balloon assistance, and it was completely filled in both the silicone model and the pig aneurysm model. In the pig subacute model, there was no recurrence after 1 month of follow-up, and it is suitable for endovascular embolization treatment of wide-necked cerebral aneurysms.

Photopolymerization has the advantages of being environmentally friendly and having wide applicability, especially when natural-product-derived monomers or photoinitiators are employed. However, it also has problems such as poor stability and limited system due to its susceptibility to interference from oxygen or impurities, as well as the narrow range of material selection. Because the purity and consistency of natural materials are more difficult to control, these problems may become even more prominent.

### 4.2 Photoisomerization

Photo-isomerization utilizes the conformational changes of specific molecules such as azobenzene and lactone under light irradiation to alter the polarity or spatial structure of the molecules, thereby destabilizing the stability of the hydrogel and ultimately achieving the photo-triggered release of drugs. Photo-isomerization does not break chemical bonds and is reversible. It can achieve a cyclic transformation between cis and trans conformations under different wavelengths of light irradiation, and thus has advantages in precise drug delivery for drug release.

**FIGURE 5:**
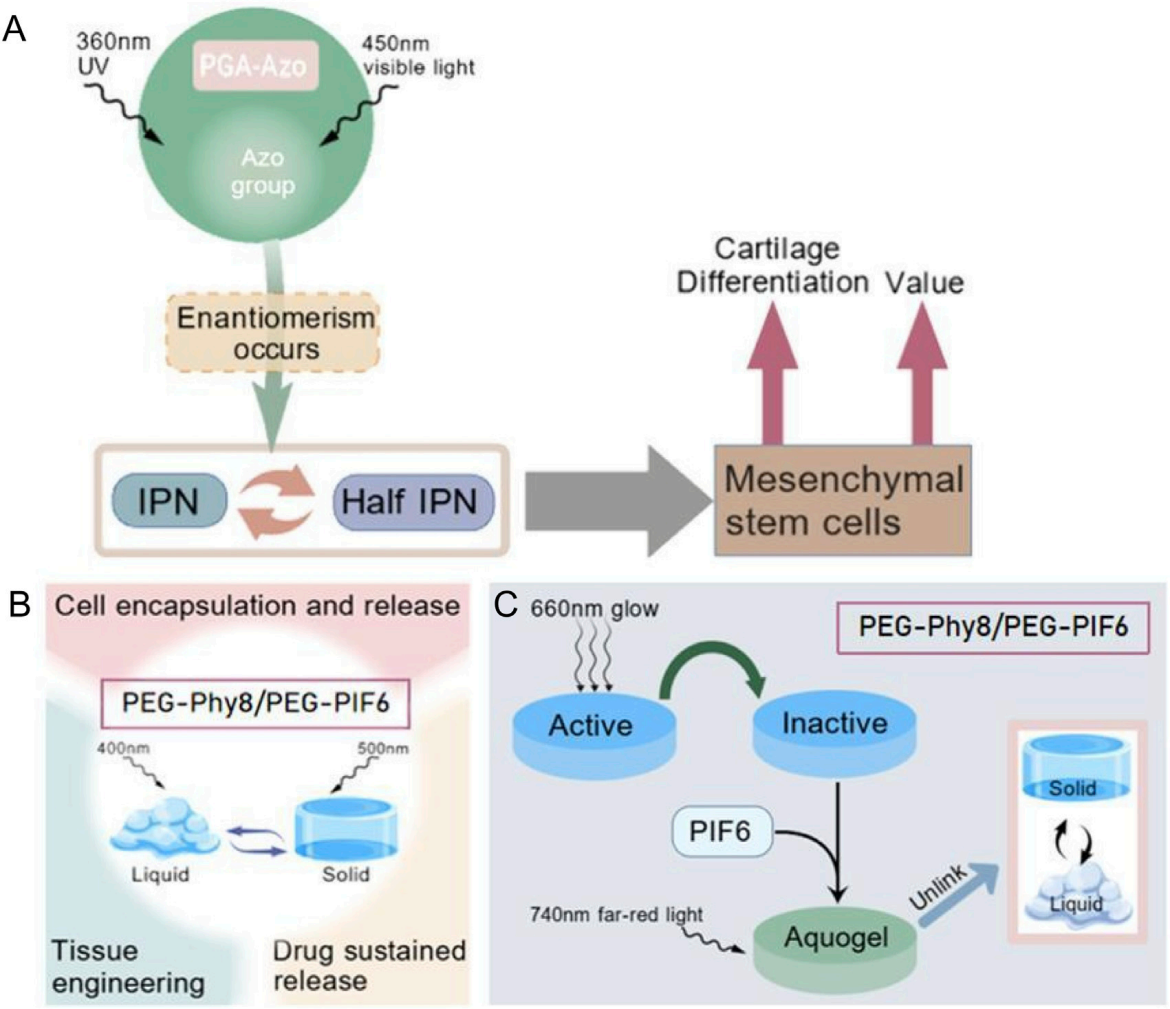
Schematic diagram of the photoisomerization principle under different reaction conditions. **(A)** PG hydrogel transitions between IPN and semi-IPN structures under 365 nm ultraviolet and 450 nm visible light irradiation (Zhang et al. 2024). **(B)** PEG-PhyB/PEG-PIF6 hydrogel undergoes reversible liquid-solid transformation under 400 nm and 500 nm light irradiation (Hoerner et al. 2023). **(C)** PEG-PhyB/PEG-PIF6 hydrogel undergoes sol-gel and sol-gel transformation under 660 nm red light and 740 nm far-red light (Narayan et al. 2025).

Zhang et al. discovered (Zhang et al., 2024) that the azobenzene groups of PGA-Azo in PG hydrogel undergo cis-trans structural changes upon exposure to 365 nm UV and 450 nm visible light, causing the PG hydrogel to transform between IPN and semi-IPN structures, which precisely regulates the proliferation and chondrogenic differentiation of BMSCs as a scaffold. The PEG-PhyB/PEG-PIF6 hydrogel prepared by Hoerner et al. (2023) can reversibly achieve liquid-solid phase transition under 400 nm and 500 nm light irradiation, and can be applied in cell encapsulation and release, tissue engineering, and drug sustained-release, etc. The PEG-PhyB/PEG-PIF6 hydrogel prepared by Narayan et al. (2025) undergoes a transformation from inactive to active state when exposed to 660 nm red light, and then forms a hydrogel through heterodimerization with PIF6; while 740 nm far-red light exposure causes the dissociation of the two, enabling the hydrogel to undergo reversible sol-gel transformation. This can be applied to the reversible extracellular matrix in microfluidic chips for the spatiotemporal-specific deposition of CHO cells through light control (Figure 5).

Photo isomerization can be carried out without the need for chemical reagents, and there is no residue of the reagents. Its feature of being recyclable makes it an important method for photo-sensitive release. However, light isomerization also has the shortcomings of instability, susceptibility to side reactions, and sensitivity to the light environment.

### 4.3 Photothermal reactions

Thermal-harmonic reaction is the combination of chromophores with photothermal conversion capabilities and thermosensitive materials. When natural-product-based molecules are involved, this advantage is more pronounced as it avoids introducing synthetic chemical residues. After absorbing specific wavelengths of light, the chromophores convert light energy into heat energy, causing the structure of the thermosensitive carrier to be disrupted, thereby achieving local drug release. This mechanism often utilizes NIR light as the excitation source, as it has strong tissue penetration and causes minimal damage to biological tissues, enabling precise drug release in deep tissues.

#### 4.3.1 Photodynamic therapy (PDT)

PDT utilizes photosensitizers that, under the action of specific light and molecular oxygen, generate reactive ROS. These reactive ROS can damage the cancer cell membrane, induce cell apoptosis, and also act as a triggering signal to regulate the release of drugs, thereby enhancing the therapeutic effect (Table 1).Among the common natural photosensitizers used in PDT, porphyrin is one of them (Wang X. et al., 2025).

**TABLE 1:** Biomedical applications driven by PDT.

Hydrogel	Main materials	Natural product	Method	Application	Reference
CS-TA/PDI hydrogel	TA\ PDI-Ala	CS	Crosslinking agent method	Treatment of melanoma	Azadikhah et al. (2021)
NIR-responsive dextran/poly (lactide) hydrogels	DEX-MA\ PpIX	Dextran, DEX	Photopolymerization method	Biological macromolecular drugs	Azadikhah and Karimi, (2022)
Injectable photosensitive supramolecular hydrogel	PVA	CS	Freeze-thaw method	wound dressing	Guerreiro et al. (2025)
CNF-DLRIHWD	CNF-PpIX/CNF-PAPB	Bagasse pulp cellulose fibre	Physical blending method	Trauma first aid, antibiotic-resistant infection wounds	Shi et al. (2022)
Silk fibroin (SF) composite hydrogel	Poly (ethylene oxide)-Poly (propylene oxide)-Poly (ethylene oxide) (PEO-PPO-PEO), Pluronic® F127 (PL127)	SA, SF, and gelatin	3D printing method	Treat skin cancer and microbial infections	Rojas et al. (2025)

Photodynamic therapy has strong targeting ability, no drug resistance, and can be repeated. However, it is also limited by the strong dependence on light and the restricted depth of tissue penetration; there are also potential risks of phototoxic reactions and long treatment cycles.

#### 4.3.2 Photothermal therapy (PTT)

PTT converts light energy into heat energy through photothermal agents. The heat causes the temperature of the hydrogel to rise, which can inhibit or even kill tumor cells. Common photosensitizers include metals such as Fe_3_O_4_ (Pan et al., 2022), Ti (You et al., 2021), and MnO_2_ (Wang et al., 2020). Some also use natural products as photosensitizers, such as ICG. PTT can precisely target tumors and achieve efficient thermal therapy using NIR light, with minimal damage to surrounding healthy tissues. Therefore, it is often used in tumor treatment in biological therapy (Pan et al., 2021). (Supplementary Table S2)

Although photothermal therapy has some limitations such as limited tissue penetration and the possibility of thermal damage, due to its minimally invasive nature, high selectivity, and relatively low side effects, it still holds great potential in combined treatment.

## 5 Quality evaluation frameworks and current limitations

### 5.1 Quality evaluation frameworks

**FIGURE 6:**
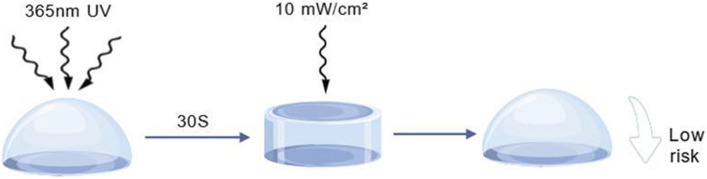
The process by which a hydrogel changes from liquid to solid state after being exposed to 365 nm ultraviolet light (10 mW/cm^2^) for 30 s (Ma et al., 2025). See Table S3.

Beyond conventional hydrogel characterization, photoinitiated hydrogels require specialized evaluations of photopolymerization efficacy and stability (Supplementary Table S3; Figure 6).

### 5.2 Current limitations

However, this system still has limitations.

Firstly, in terms of biocompatibility, biocompatibility testing, as an indispensable routine test, usually includes aspects such as cell tests, *in vivo* tests, and *in vitro* tests. Li et al. (2018), by injecting hydrogels into the subcutaneous hematological parameters of mice, showed that there was no significant difference between the mice injected with hydrogels and PBS, and the degradation rate in tumors was faster than that in normal tissues. Wang et al. (2026) found that the proliferation ability of BMSCs showed a trend of first increasing and then decreasing with the addition amount of HA-MXene. When the concentration of HA-MXene reached 500 ppm, the OD value decreased significantly and the number of cells decreased sharply, showing certain cytotoxicity. Within the suitable concentration range of HA-MXene, the ATAM-1 hydrogel coating has certain biocompatibility. Rosales et al. (2018) encapsulated NIH 3T3 fibroblasts with 6wt% Azo_28_HA/CD_28_HA hydrogel. The 1-day survival rate was approximately 90%, and a high survival rate remained within 3 days. The cell morphology is round. The swelling of the hydrogel leads to a slight decrease in cell density, but there is no obvious toxicity. Although some studies have investigated biocompatibility, in accordance with ICH guidelines, most photosensitive hydrogels undergo photosafety evaluations of drugs as required. Most studies lack a complete evaluation of this part, thus failing to truly reflect the practical application level of photosensitive hydrogels and greatly restricting the development of hydrogels in future clinical applications.

Secondly, although current research on light-responsive drug delivery systems has explored drug release behavior in both dark and light conditions, most experiments have not systematically studied the differences in drug use under different lighting conditions.

In addition, in terms of reactive oxygen species (ROS) detection, although there is a preliminary quantitative analysis, more precise methods are needed to ensure the safety of *in vivo* application.

Meanwhile, key transformation links such as GMP-scale extraction processes, light stability, storage conditions, batch consistency, and regulatory pathways have rarely been covered. It is necessary to establish a complete research system to support large-scale production.

Finally, most existing studies focus on short-term wound healing and tumor suppression effects. However, the long-term nature of tumor treatment requires drugs to be released for a long time, safely and controllability, which means that long-term and repeated dosing strategies still need to be strengthened. Moreover, although it is known that the sources, extraction methods and batch differences of natural polymers may affect material quality, there is still a lack of systematic assessment of the specific influencing mechanisms of these factors at present.

## 6 Conclusions and future research perspectives

This article systematically reviews the application achievements of natural-product-based light-responsive hydrogels in aspects such as light-responsive properties and preparation technologies of light-responsive hydrogels. It includes the advantages of natural products-based light-responsive hydrogels, preparation methods, applications based on photo-responsive mechanisms, and evaluation systems. Natural products-based light-responsive hydrogels, as functional materials that combine natural active components with photo-responsive properties, have shown unique potential in biomedicine (Fan et al., 2024) and tissue engineering (Salahuddin et al., 2021) fields in recent years.

Based on the above research status, its actual development needs to establish a strict control system in multiple links. In the raw material quality control stage, it is necessary to clearly define the species, origin and harvest time of natural products and establish batch files. Through standardized extraction, separation and purification processes, the chemical composition fluctuations between batches should be reduced. At the same time, quantitative analysis of key components of raw materials should be conducted to ensure compliance. Ultimately, batch consistency and traceability management should be achieved. During the gelation process, the degree of modification (DS), as a core process parameter, requires the precise determination of the number of photosensitive groups on natural molecules, as it directly determines the subsequent photopolymerization speed and the final gel performance. During the photo-curing process, low-cytotoxic photoinitiators should be given priority and the safe residual concentration should be determined. At the same time, the light wavelength, intensity and time should be strictly regulated and monitored. These parameters play a decisive role in the crosslinking density, network uniformity and cell survival rate of the hydrogel. During the final product evaluation stage, it is necessary to test physical and chemical properties such as mechanical strength, swelling rate, and degradation rate to match the target application scenarios. The cytotoxicity, sensitization, and *in vivo* immune response should be evaluated to ensure biological safety. At the same time, the drug’s controlled-release ability, cell growth support performance, and the inherent antibacterial and anti-inflammatory special activities of the natural product should be verified to ensure that the biological functionality meets the standards.

The development of light-responsive hydrogels based on natural products is still constrained by multiple practical bottlenecks and has not yet been widely applied. Judging from the current research and application status, the core issues are concentrated in three aspects. First of all, the application scenarios are limited. Due to their reliance on light conditions, existing hydrogels are mainly used in simple scenarios such as *in vitro* antibacterial and local superficial tissue repair. There have been almost no breakthroughs in complex fields such as organ regeneration and precise drug delivery. Especially in the field of tumor treatment, tumor treatment usually requires long-term administration of drugs. However, current research in this field is limited to short-term administration patterns and has not yet established a mechanism for long-term repeated administration, ignoring the effects brought about by long-term administration. Moreover, even in other applications such as wound dressings, the research on the drug delivery mechanism only focuses on precise site administration, neglecting the effects on the overall condition and immune regulation. The second issue is the insufficient capacity for deep processing. The existing photosensitive systems are highly dependent on the penetration depth of light. However, visible light has poor penetration in biological tissues, making it difficult to establish effective light response conditions in deep lesion areas, which limits the application of deep tissue treatment. Ultraviolet light, as the leading cause of skin cancer, should be restricted in its direct application on the skin or its usage duration should be controlled to ensure its safe use. Near-infrared is expected to be applied. Research on the enhancement of NIR has gradually become prominent. It is expected that NIR ranging from several millimeters to several centimeters is suitable for medical imaging and treatment related to deep tissues, while MIR ranging from micrometers to several millimeters is applicable to surface-level medical applications. The two complement each other and support a variety of non-invasive medical technologies (Zohuri, 2025; Wang et al., 2022). Third, the R&D efficiency is relatively low. Although natural products are abundant and can significantly enhance the biocompatibility of hydrogels, their application as photosensitive groups remains limited. Currently, regarding the regulation mechanisms of the concentration of active components and biological efficacy of natural products through the optimization of collection time and extraction process, no systematic and complete theoretical framework and technical specifications have been established in related research. This, to a large extent, restricts the subsequent research and development process as well as practical application transformation of natural product-based hydrogels. Further enhance the *in vivo* utilization rate of materials and reduce the rejection reaction of the human body to traditional photoreactive hydrogels. However, at present, the preparation of this kind of hydrogel requires a large number of repeated experiments for screening and optimization, which consumes too much manpower and time. It has restricted the innovation and research process of new hydrogels.

Future development needs to enhance efficiency through technological innovation and empowerment The preparation end can integrate technologies such as electrospinning (Lee and Song, 2023), 3D printing (He et al., 2022) and *in situ* polymerization (Wang H. et al., 2025) to precisely control the microscopic and macroscopic structures of hydrogels, thereby endowing them with multiple functions such as diagnosis, treatment and monitoring. At the same time, it provides support for the efficient introduction of photosensitive groups based on natural products. For instance, electrospinning technology can construct bionic microchannel networks to facilitate tissue regeneration. 3D printing can adapt to complex application scenarios. The evaluation system needs to improve the systematic assessment dimensions such as biocompatibility and utilization rate, and fill the gaps in precise detection of photosensitive group content from natural product sources and in-depth assessment of tissue safety after light exposure. The application of artificial intelligence technology can solve the problem of high-cost screening, such as reducing the number of experimental iterations through data-driven design strategies, and accelerating and improving the efficiency of new material research and development (Zhang D. et al., 2025; Yang et al., 2018).

With the continuous development of AI, it has been applied in the summary and development of hydrogels (Meijer et al., 2025). In the realm of natural product - based light - responsive hydrogels, AI can play a crucial role. In the process of preparing these hydrogels, AI can screen materials including natural products and photosensitizers, select appropriate material ratios and concentrations, and simulate the preparation results and application outcomes (Gangwal and Lavecchia, 2025). For instance, Jiang et al. (Jiang et al., 2025) proposed the development of an AI-guided AMP-hydrogel-Designer platform. Through AI techniques such as generative pre - training, prompt tuning, knowledge distillation, and reinforcement learning, they designed the highly efficient and broad-spectrum antimicrobial peptide AK15 containing thiol groups. The prepared hydrogel has a bactericidal rate of over 99.99%, and the 6-day wound closure rate in the rat dynamic wound model reaches 99.5%. Neto et al. (2025) utilized the Google Teachable Machine 2.0 platform to train an AI model for classifying and predicting histological images. They analyzed the model’s recognition accuracy through a confusion matrix to distinguish the properties of stretched states and different formulations of hydrogels, which is valuable for evaluating natural product - based light - responsive hydrogels.

By studying relevant databases, AI can also provide services for data analysis related to these hydrogels. Chen et al. (2025) utilized AI to analyze the spectral information of sweat biomarkers captured by hydrogels, thereby achieving precise diagnosis of lung cancer treatment, demonstrating AI’s potential in diagnostic applications of hydrogel - based systems. In addition, AI can learn the relevant information of known natural products through training, and then be capable of predicting and discovering new natural products (Saldívar-González et al., 2022). This can help expand the range of natural products used in light - responsive hydrogels. Shen et al. (2025) utilize AI algorithms to model and optimize the existing experimental data of hydrogels, which can accelerate the development process of natural product - based light - responsive hydrogels.

Specifically, artificial intelligence can be used to construct light transmission models in biological tissues (Li et al., 2024). By combining the optical properties of natural - product photosensitizers, it can simulate the deep photo - responsive efficiency under different light conditions and quickly screen out suitable material combinations for light control; at the same time, artificial intelligence can assist in designing photodynamic systems and learn to predict the interactions between natural - product derivatives and stimulus - responsive polymers.

In summary, natural product-based light-responsive hydrogels need to be developed based on existing problems. By integrating multiple preparation methods and leveraging artificial intelligence technology to solve the bottlenecks in efficiency and performance, and improving the entire research and evaluation system from preparation to evaluation. The value of this review lies in elaborating on the method selection during the preparation process of hydrogels and analyzing the potential development directions. Based on this, it is hoped that it can provide guidance for exploring the future innovative directions of hydrogels based on new natural products.
