# Supplementary material for: Preparation and biomedical application of light-responsive hydrogels based on natural products
Source: Front Pharmacol. 2025 Dec 3;16:1714907. doi: 10.3389/fphar.2025.1714907 (PMC12739651; doi:10.3389/fphar.2025.1714907)
Supplement: Supplementary file 1 [file Table1.docx]

Supplementary Material

# Supplementary Tables

TABLE 2 Biomedical Applications Driven by PTT

| Hydrogel | Main materials | Natural product | Method | Application | Reference |
| --- | --- | --- | --- | --- | --- |
| SCP/LM bilayer hydrogel actuator | N-Isopropylacrylamide (NIPAAm)/ AA/ 1-Pyrene methyl acrylate (Py-Ac) | Sea Cucumber Peptide（SCP） | formed by UV polymerization | soft robotics and biomedical field | (C. Zhang et al. 2025) |
| QCH/PBₙ | Prussian blue nanoparticles | Chitosan | Free radical polymerization method | Wound on the surface of the skin | (Han et al. 2020) |
| RL-Zc Hydrogel | Succinoglycan riclin | Zinc phthalocyanine（Zc） | cross-linking by covalent bonding | Infection wound repair | (Yang et al. 2025) |
| MCP@ZIF-8 | MoS₂、Ce6 | CMCS | Crosslinking agent method | Treatment of wound infected with drug-resistant bacteria | (W. Zhang et al. 2025) |
| BP@Hydrogel | PEGylated BPNSs | low–melting-point agarose、doxorubicin，DOX | Physical method | Accurate cancer treatment | (Qiu et al. 2018) |
| I/G-LPMSN@HAMA | I/G-LPMSN | HA | Chemical photopolymerization crosslinking method | Bacterial clearance within the tumor, treatment of osteosarcoma, etc. | (Hu et al. 2025) |
| Near-infrared light-responsive collagen hydrogel | Gold nanorods (GNRs), liposome materials, growth factors | Collagen from the tail of a rat | Physical embedding and chemical coupling | Promote angiogenesis, etc. | (Nazemidashtarjandi et al. 2024) |
| MPS-TA-MXene/PNIPAM-PAM composite hydrogel | MXene nanosheets, lithium chloride (LiCl) | Tannic acid (TA) | Crosslinking agent method | Underwater exploration and archaeology, flexible electronics and intelligent robots, etc. | (Li et al. 2024) |
| SA/DOX@hydrogel | Ag-doped SnS? nanocylinders (SA) | Adriamycin hydrochloride (DOX-HCl) and agarose | Physical blending method | Cancer treatment | (Yang et al. 2023) |
| oxidative stress-enhancing hydrogel system（EOH） | Cu-Hemin-Au | agarose | hydrothermal method | tumor inhibition | (Liu et al. 2023) |
| MoS₂@PDA@Ag/PVA | Molybdenum disulfide (MoS₂) nanoparticles, silver nanoparticles (AgNPs) | Polydopamine (PDA) | Hydrothermal method | Treat bacterial-infected wounds | (Yan et al. 2022) |
| Nb₂C-Arg/Gel Composite Multifunctional Hydrogel | Nb₂C NSs、Arg | SA、  CaCl₂ | ion exchange method | Treatment of infected wounds caused by drug-resistant bacteria | (Liu et al. 2025) |

TABLE 3 Quality Evaluation Frameworks

| Evaluation Dimension | Core Content | Detection Method | Result | Reference |
| --- | --- | --- | --- | --- |
| Cross-linking Property | Cross-linking Density | Electron Fiber Scanning | When the concentration of HA-NB is between 2% and 4%, the cross-linking degree of the hydrogel network increases. | (W. Zhu et al. 2025) |
|  | Cross-linking Rate | 365nm UV Irradiation | The hydrogel changes from liquid to solid state within 30 seconds. | (Ma et al. 2025)  (FIGURE 6) |
| Mechanical Property | Degradation Rate | LC-MS | The main degradation products are bilirubin and nitrosophenol. The free volume in the sample increases with the extension of irradiation time, and the maximum concentration corresponds to a degradation efficiency of 73%. | (Claaßen et al. 2018) |
|  | Structural and Performance Stability | Near-infrared Irradiation | In 5 cyclic tests of the py-0 sample, the deformation angle and recovery ability are stable. | (Zhao et al. 2024) |
|  | Rheological Property | Different Power UV Irradiation | It has different mechanical properties. When the light intensity increases, the viscosity of the sample also increases. | (Dobashi et al. 2021) |
| Biocompatibility | Cytotoxicity | Dark State vs Light State Comparison | Under no-light conditions, GQDs have minimal impact on DOK cell viability, showing extremely low dark toxicity. After light irradiation, free GQDs show high cytotoxicity within 12 hours; G@GQD hydrogel shows a continuous and prolonged killing effect. | (Lv et al. 2025) |
|  | Tissue Compatibility | ROS and Inflammatory Factor Detection | After two weeks of treatment with HTONPd@SelHVs, compared with normal mice without PU wound surface, HTONPd@SelHVs has no significant impact on the levels of ROS and various inflammatory factors. | (J. Zhu et al. 2025) |
|  | Hemocompatibility | Waltham, USA | The hemolysis rate of all hydrogel patches is less than 2%. | (Dai et al. 2026) |
| Functional Property | Drug Release Efficiency | Near-infrared Drug Release Evaluation | The drug release rate of ciprofloxacin under irradiation is significantly higher than that without irradiation. | (Yang et al. 2020) |
|  | ROS Scavenging | γ-ray Irradiation | Compared with the HF group and the control group, the PHF@Res-2 group significantly reduces the DCFH-DA fluorescence intensity in L929 cells irradiated by γ-rays. | (Shen et al. 2025) |

**REFERENCE:**

Claaßen, Christiane, Marc H. Claaßen, Fabian Gohl, Günter E. M. Tovar, Kirsten Borchers, and Alexander Southan. 2018. “Photoinduced Cleavage and Hydrolysis of *o* ‐nitrobenzyl Linker and Covalent Linker Immobilization in Gelatin Methacryloyl Hydrogels.” *Macromolecular Bioscience* 18 (9): 1800104. https://doi.org/10.1002/mabi.201800104.

Dai, Suyang, Lingchen Mao, Xiongwei Chen, et al. 2026. “A Heterogeneous Hydrogel Patch with Mechanical Activity and Bioactivity for Chronic Diabetic Wound Healing.” *Biomaterials* 324 (January). https://doi.org/10.1016/j.biomaterials.2025.123531.

Dobashi, Yuta, Jerry C. Ku, Christopher Pasarikovski, et al. 2021. “Dynamically Tunable Intravascular Catheter Delivery of Hydrogels for Endovascular Embolization.” *MRS Advances* 6 (3): 66–71. https://doi.org/10.1557/s43580-021-00047-8.

Han, Donglin, Yuan Li, Xiangmei Liu, et al. 2020. “Rapid Bacteria Trapping and Killing of Metal-Organic Frameworks Strengthened Photo-Responsive Hydrogel for Rapid Tissue Repair of Bacterial Infected Wounds.” *Chemical Engineering Journal* 396 (September): 125194. https://doi.org/10.1016/j.cej.2020.125194.

Hu, Hanyin, Zhuoming Xu, Jintao Liu, et al. 2025. “Light-Responsive Hydrogel with Nano-Microstructures for Bacteria Eradication and Tumor Therapy.” *ACS Applied Nano Materials* 8 (12): 6242–54. https://doi.org/10.1021/acsanm.5c01116.

Li, Yongji, Zhongyuan Tian, Changjun Li, Zheng Li, Zhong-Zhen Yu, and Dan Yang. 2024. “Bionic Light-Responsive Hydrogel Actuators with Multiple-Freedom Motions in Water Environments.” *Nano Energy* 130 (November): 110130. https://doi.org/10.1016/j.nanoen.2024.110130.

Liu, Mi, Weidan Zhao, Denghao Li, et al. 2025. “A NIR-II Laser-Triggered Nb_2_ C-Arg/Gel Composite Multifunctional Hydrogel with Photothermal, ROS Scavenging, and NO Release for Infected Wound Healing.” *Langmuir* 41 (26): 17104–16. https://doi.org/10.1021/acs.langmuir.5c01761.

Liu, Zeming, Hongbo Chen, Chunyu Huang, and Qinqin Huang. 2023. “A Light-Responsive Injectable Hydrogel with Remodeling Tumor Microenvironment for Light-Activated Chemodynamic Therapy.” *Macromolecular Bioscience* 23 (1): 2200329. https://doi.org/10.1002/mabi.202200329.

Lv, Zhengzheng, Xin Xia, Peisheng Cao, et al. 2025. “Overcoming the Drug Retention Barrier with Photosensitive Hydrogel for Sustained Photodynamic Therapy of Oral Leukoplakia.” *Chinese Chemical Letters*, April, 111282. https://doi.org/10.1016/j.cclet.2025.111282.

Ma, Peifen, Jianlong Da, Guanghui Zhao, et al. 2025. “Injectable Light-Responsive Hydrogel Dressing Promotes Diabetic Wound Healing by Enhancing Wound Angiogenesis and Inhibiting Inflammation.” *Polymers* 17 (5): 607. https://doi.org/10.3390/polym17050607.

Nazemidashtarjandi, Saeed, Bryce Larsen, Kristie Cheng, et al. 2024. *Near-Infrared Light-Responsive Hydrogels for on-Demand Dual Delivery of Proangiogenic Growth Factors*.

Qiu, Meng, Dou Wang, Weiyuan Liang, et al. 2018. “Novel Concept of the Smart NIR-Light–Controlled Drug Release of Black Phosphorus Nanostructure for Cancer Therapy.” *Proceedings of the National Academy of Sciences* (United States) 115 (3): 501–6. https://doi.org/10.1073/pnas.1714421115.

Shen, Jintao, Wencheng Jiao, Junzhe Yang, et al. 2025. “In Situ Photocrosslinkable Hydrogel Treats Radiation-Induced Skin Injury by ROS Elimination and Inflammation Regulation.” *Biomaterials* 314 (March): 122891. https://doi.org/10.1016/j.biomaterials.2024.122891.

Yan, Pengfei, Mengya Li, Jie Liu, Yadi Hu, and Keyong Tang. 2022. “MoS2@PDA@Ag/PVA Hybrid Hydrogel with Excellent Light-Responsive Antibacterial Activity and Enhanced Mechanical Properties for Wound Dressing.” *Macromolecular Materials and Engineering* 307 (2): 2100654. https://doi.org/10.1002/mame.202100654.

Yang, Jun, Zhen Sun, Qingqing Dou, et al. 2023. “NIR-Light-Responsive Chemo-Photothermal Hydrogel System with Controlled DOX Release and Photothermal Effect for Cancer Therapy.” *Colloids and Surfaces, A: Physicochemical and Engineering Aspects* 667 (June): 131407. https://doi.org/10.1016/j.colsurfa.2023.131407.

Yang, Na, Ming Zhu, Guochao Xu, Ning Liu, and Cong Yu. 2020. “A Near-Infrared Light-Responsive Multifunctional Nanocomposite Hydrogel for Efficient and Synergistic Antibacterial Wound Therapy and Healing Promotion.” *Journal of Materials Chemistry B* 8 (17): 3908–17. https://doi.org/10.1039/d0tb00361a.

Yang, Yunxia, Hongmei Zhang, Xueqing Zhang, et al. 2025. “A Succinoglycan-Riclin-Zinc-Phthalocyanine-Based Composite Hydrogel with Enhanced Photosensitive and Antibacterial Activity Targeting Biofilms.” *Gels* 11 (8): 672. https://doi.org/10.3390/gels11080672.

Zhang, Congyu, Qi Zheng, Cui Li, et al. 2025. “A Multifunctional Electrochemical Biosensor Based on Near-Infrared Light-Responsive Hydrogel for In Vivo Recording and Modulation.” *Analytical Chemistry* 97 (23): 12164–70. https://doi.org/10.1021/acs.analchem.5c00640.

Zhang, Weiwei, Yongqi Yang, Xuanjun Zhang, et al. 2025. “Research on the Combined Antibacterial and Wound-Healing Effects of Oxygen-Carrying Hydrogel Photodynamic and Photothermal Therapy-Targeting Biofilms.” *ACS Applied Materials & Interfaces* (United States) 17 (36): 50404–16. https://doi.org/10.1021/acsami.5c11868.

Zhao, Xinyu, Jinqiang Jiang, Zhongwen Liu, Zhaotie Liu, and Guo Li. 2024. “Thermal and Near-Infrared Light-Responsive Hydrogel Actuators with Spatiotemporally Developed Polypyrrole Patterns.” *ACS Applied Materials & Interfaces* 16 (7): 9286–92. https://doi.org/10.1021/acsami.3c17885.

Zhu, Jiawei, Ting Chen, Wei Fang, et al. 2025. “Local Visible-Photocatalytic Production of Hydrogen and Warm Heat for Combination Treatment of Pressure Ulcer.” *Advanced Science (Weinheim, Baden-Wurttemberg, Germany)* (Germany) 12 (33): e03185. https://doi.org/10.1002/advs.202503185.

Zhu, Wei, Han Wang, Bin Feng, et al. 2025. “Self-Healing Hyaluronic Acid-Based Hydrogel with miRNA140-5p Loaded MON-PEI Nanoparticles for Chondrocyte Regeneration: Schiff Base Self-Assembly Approach.” *Advanced Science (Weinheim, Baden-Wurttemberg, Germany)* (Germany) 12 (1): e2406479. https://doi.org/10.1002/advs.202406479.
